# Histology of Interstitial Lung Disease in Common Variable Immune Deficiency

**DOI:** 10.3389/fimmu.2020.605187

**Published:** 2020-11-20

**Authors:** Fatima Dhalla, Dylan J. Mac Lochlainn, Helen Chapel, Smita Y. Patel

**Affiliations:** ^1^ Department of Clinical Immunology, John Radcliffe Hospital, Oxford University Hospitals NHS Foundation Trust, Oxford, United Kingdom; ^2^ Developmental Immunology, MRC Weatherall Institute of Molecular Medicine, University of Oxford, Oxford, United Kingdom; ^3^ Primary Immunodeficiency Unit, Nuffield Department of Medicine and National Institute for Health Research Oxford Biomedical Research Centre, University of Oxford, Oxford, United Kingdom

**Keywords:** common variable immune deficiency, interstitial lung disease, histology review, literature analysis, primary immune deficiencies

## Abstract

Interstitial lung disease (ILD) is an important non-infectious complication in several primary immune deficiencies. In common variable immune deficiency (CVID) it is associated with complex clinical phenotypes and adverse outcomes. The histology of ILD in CVID is heterogeneous and mixed patterns are frequently observed within a single biopsy, including non-necrotising granulomatous inflammation, lymphoid interstitial pneumonitis, lymphoid hyperplasia, follicular bronchiolitis, organizing pneumonia, and interstitial fibrosis; ILD has to be differentiated from lymphoma. The term granulomatous-lymphocytic interstitial lung disease (GLILD), coined to describe the histopathological findings within the lungs of patients with CVID with or without multisystem granulomata, is somewhat controversial as pulmonary granulomata are not always present on histology and the nature of infiltrating lymphocytes is variable. In this mini review we summarize the literature on the histology of CVID-related ILD and discuss some of the factors that may contribute to the inter- and intra- patient variability in the histological patterns reported. Finally, we highlight areas for future development. In particular, there is a need for standardization of histological assessments and reporting, together with a better understanding of the immunopathogenesis of CVID-related ILD to resolve the apparent heterogeneity of ILD in this setting and guide the selection of rational targeted therapies in different patients.

## Introduction

Common variable immune deficiency (CVID) is the most common of the primary immunodeficiency (PID) syndromes with a prevalence of 1 in 25,000 and 50,000, depending on the population (1, 2). It is characterized by low serum levels of IgG, IgA, and/or IgM, and poor specific antibody production (3). There is no definitive diagnostic test, so diagnosis requires the exclusion of secondary hypogammaglobulinaemia, combined immune defects, and, where appropriate, Mendelian disorders (4, 5). Up to 70% of patients suffer with variable non-infectious complications reflecting broader immune dysregulation, including autoimmunity, most commonly autoimmune cytopaenias; lymphocytic infiltration and/or granulomatous inflammation which can affect the lungs, gastrointestinal tract, spleen, skin or liver; or malignancy, in particular lymphoma (6, 7). Importantly, while bacterial infections are significantly reduced by adequate replacement therapeutic IgG, disease-related complications are not, but are associated with substantially increased mortality (7–9).

Respiratory tract pathology is a major contributor to impaired quality-of-life (10). Bacterial sinopulmonary infections are often the presenting feature, most frequently caused by *Haemophilus influenzae* or *Streptococcus pneumoniae* (11, 12). Recurrent and/or severe lower respiratory tract infections, particularly pneumonia, lead to bronchiectasis with an overall estimated prevalence of 30-35% among CVID patients, which, when present in isolation, does not contribute to increased mortality (8, 11–14). Interstitial lung disease (ILD), on the other hand, probably occurs due to immune dysregulation and/or viral infection rather than as a consequence of bacterial infection (7, 15, 16), and occurs alongside other disease-related complications, and shortens survival (7–9, 16). More rarely, the lungs can be the location for extranodal lymphomas, particularly B-cell non-Hodgkin’s lymphomas or MALToma (7, 17–20).

## Interstitial Lung Disease in Common Variable Immune Deficiency

### Clinical Significance of CVID-Related ILD

ILD is among the more frequent non-infectious complications of CVID, reported in 15%–60% of patients (7, 9, 14, 21–23). Clinical symptoms and high-resolution computed tomography (HRCT) findings of ILD can appear before or after CVID diagnosis (24, 25). The pathogenesis of CVID-related ILD is presumed to be unrelated to bacterial infections because it can be seen in the absence of bronchiectasis and is not significantly associated with a history of pneumonia (21). Patients with ILD have distinct clinical and immunological phenotypes in keeping with immune dysregulation, in contrast to those without ILD or those with bronchiectasis alone (6, 9, 14, 16, 21, 26, 27). Furthermore, there is no current histological or molecular evidence for chronic bacterial, EBV or CMV viral infections as triggers for inflammation (16, 28–30), though granulomas in other PIDs, such as those with DNA repair defects, show evidence of vaccine derived rubella virus (31). Other related complications, including splenomegaly, autoimmune cytopaenias, persistent lymphadenopathy and lymphoproliferation, but not necessarily granulomata, occur more frequently in patients with CVID-related ILD, supporting at least a role for intrinsic immune dysregulation driving these varied features (6, 9, 16, 21, 27, 32, 33).

Since CVID-related ILD causes significant morbidity, can be progressive and contributes to mortality, there is urgent need for effective treatments (8, 9, 34, 35). Because the mechanism(s) underlying CVID ILD have not been elucidated, immunosuppressive treatments have been tried with varying success, including corticosteroids, ciclosporin, methotrexate, sirolimus, cyclophosphamide, hydroxychloroquine, anti-TNF agents, mycophenolate mofetil, abatacept, rituximab and azathioprine (16, 34, 36–38). Corticosteroids are often used first-line, however, response may be short-lived or incomplete, there are significant side effects associated with protracted use and a proportion of patients are refractory (16, 34, 36, 39). Success with Rituximab, both in combination with azathioprine or mycophenolate mofetil, and as monotherapy, has been reported although controlled trials and long-term outcome data are lacking (40–43). Elevated levels of B-cell activating factor (BAFF), a cytokine that promotes the maturation and survival of B-cells, within the serum and lungs of patients with CVID-related ILD levels drives B-cell hyperplasia and may account for disease progression in a small proportion of patients (15) with invasive B cells in inappropriate germinal centers (28, 44).

### Nomenclature

Various terminologies are used for CVID-related ILD, reflecting a lack of consensus regarding the naming of this complication and its heterogeneous nature (45). Lymphoid interstitial pneumonitis was first reported in patients with antibody deficiency in 1973 (46). Since then, various histopathological entities have been reported within lung biopsies of CVID ILD patients, from those caused by polyclonal lymphocytic inflammation to well-formed granulomata, organizing pneumonia, or pulmonary fibrosis, often with mixed pathology within individual patient biopsies (7, 9, 16, 27, 33, 35, 44). “Granulomatous-lymphocytic interstitial lung disease” (GLILD), first proposed in 2004, is often used as an overarching term to describe CVID ILD with lymphocytic infiltrates and/or granulomata (9, 45). However, the accuracy of this term has been called into question. Since not all patients have pulmonary granulomata, it does not fully capture the heterogeneity of the histopathology and similar histological patterns fulfilling a GLILD diagnosis are found in non-CVID PIDs (33, 47).

### Investigations for CVID-Related ILD

Non-invasive investigations for CVID-related ILD include elevated serum IgM, decreased class-switched memory B-cells and absolute/relative numerical abnormalities of T-cell populations (15, 16, 34, 35, 48). Alongside rising IgM levels, BAFF, soluble IL-2 receptor and β2microgloblin have also been proposed as serum biomarkers for disease activity (15, 34, 49). Lung function tests, particularly the diffusion capacity for carbon monoxide (DLCO), are useful in monitoring for disease progression and response to treatment, but can lack the sensitivity required for diagnosis, particularly early in the disease course (14, 28, 34, 35, 37). HRCT is highly sensitive for the detection of CVID ILD, including at an early stage before symptoms or abnormal pulmonary function have developed (14, 33, 34). Radiographic findings are mixed and include lymphadenopathy, ground glass opacification, nodularity, septal thickening and consolidation (21, 33, 50). The use of CT combined with positron emission technology (PET) has also been reported as useful to identify sites of active disease, guide biopsy sampling, and monitor response to treatment (41). In selected cases, particularly, but not restricted to, pediatric presentations, genetic testing may be warranted. For example, patients with mutations in *CTLA4*, *LRBA, TACI, KMT2D, XIAP, RAG1*, and *NFKB1* have been found within so called “CVID” cohorts, and ILD is a common feature of other monogenic PIDs (34, 39, 51–57). A molecular diagnosis enables other therapeutic approaches such as CTLA-4 fusion proteins abatacept and belatacept for the inflammatory associations of CTLA-4 and LRBA deficiency (58, 59). Invasive investigations include assessment of bronchoalveolar lavage fluid for infection and lymphocyte phenotyping, often used to avoid possible complications of biopsy (60), or biopsy of lung tissue under imaging for histopathological assessment.

### Importance of Histopathological Assessment of Lung Tissue

Histological assessment of affected lung tissue is essential if features of ILD are present on HRCT. Imaging alone is not sufficient because radiographic patterns of parenchymal lung disease do not correlate with pathological features (33). It has been suggested that tissue from more accessible organs could be used in lieu of lung biopsy (34); however, patients with granulomata at other sites do not necessarily display granulomata within areas of ILD, indicating that other organs do not necessary serve as a proxy for the lung (33). Importantly, histological assessment contributes to the exclusion of differential diagnoses including infection and lymphoma and can provide prognostic information, since interstitial fibrosis has been associated with poorer outcomes (7, 17–20, 33). Currently, it is common practice to subject lung biopsy specimens to hematoxylin and eosin (H&E) staining, immunohistochemical staining for CD3, CD4, CD20/19 and EBV and CMV viral infections (37, 44). Understanding the pathological processes at play and the phenotype of infiltrating immune cells can help rationalize the selection of therapeutics used for CVID ILD (40–43).

We have reviewed the published literature of large series (>10 cases) for detailed histological findings of CVID ILD, the most recent being Larsen et al. (46). It is not always possible to know which patients were included in previous reports so only the most recent from each center is used unless marked (**Table 1**). Variations including the methods used for both biopsy and reporting are discussed in Section 4.

**Table 1:** Histological lung biopsy findings from common variable immune deficiency (CVID) patients reported in the literature.

Histological findings									
Publication (Ref)	Number of CVID patients with lung biopsies	Granulomata n (%)	Pulmonary Lymphoid Hyperplasia				Organizing pneumonia	Pulmonary Fibrosis	
			*Interstitial inflammation*	*(Peri)bronchial inflammation*	*Lymphocytic infiltration*	*Lymphoid hyperplasia*		*Fibrosis*	*Remodeling*
Rao et al.* (44)	16	15 (93%)	16 (100%)	16 (100%)	NS	NS	14 (87%)	12 (75%)	6 (37%)
Patel et al. (33)	19	1 (5%)	11 (58%)	7 (37%)	15 (79%)	NS	6 (32%)	8 (42%)	3 (16%)
Maglione et al. (21)	12	3 (25%)	4 (33%)	4 (33%)	2 (17%)	4 (33%)	4 (33%)	4 (33%)	NS
Larsen et al. (47)	34	23 (68%)	12 (35%)	22 (65%)	NS	10 (29%)	25 (71%)	1 (3%)	NS
Verbsky et al.* (61)	34	31/34 (91%)	NS	33/34 (97%)	33/34 (97%)	NS	30/34 (88%s	13/34 (32%)**	NS

Only publications with sufficient histological detail were included; single case histories or small studies (less than 10) are not included. Rao et al. (44) and Patel et al. (33) reported their findings in similar terms, but these varied in other publications. Efforts were made to group similar findings on the basis on similar histological terms in these instances. Where detail for a given finding was not specified (NS), this is also indicated. *Where the inclusion of previously published cases in a paper could not be completely excluded. ** on CT not reported on histology.

## Histological Patterns of ILD in CVID

The histological abnormalities reported in CVID ILD vary and overlap extensively. Similar patterns can also be found in numerous other lung diseases, making diagnosis challenging (44). Using a similar structure as Rao et al. (44), we summarize the commonly reported lung biopsy findings, each of which we discuss in turn (**Table 1**).

### Granulomata

The granulomata reported in CVID ILD can vary from poorly- to well-circumscribed, with an apparent predilection for the former (28, 33, 44). Non-infectious CVID granulomatous lung disease shares some similar histological features with sarcoidosis and hypersensitivity pneumonitis; thus, clinical and radiological correlation is important in distinguishing these conditions (44, 62). “Poorly-formed granulomata” have been found within areas of pulmonary lymphoid hyperplasia and are difficult to define, as these are very subjective; additionally, granulomata can be found throughout the lung parenchyma (28, 44). It is worth re-emphasizing that granulomata are not reported in all cases of CVID-related ILD, with frequencies ranging from 0-94% depending on the individual study (**Table 1**) (7, 33, 44, 47). This suggests that there may be more than one pathological process in CVID-ILD (33, 47) and that the generalized use of overarching term “GLILD” to refer to all CVID-related ILD can be misleading.

### Pulmonary Lymphoid Hyperplasia

Lymphoid proliferation has been designated as the “cardinal” feature of CVID ILD, and different patterns of pulmonary lymphoid hyperplasia (PLH) have been described, including follicular bronchiolitis, lymphocytic interstitial pneumonitis (LIP), lymphocytic infiltrates, and nodular lymphoid hyperplasia (28, 38, 40, 44, 47). In one case series where severity was assessed, PLH tended to be toward the moderate to severe end of the spectrum, with peribronchiolar and interstitial lymphocytic inflammation (44). These patterns often occur together and are rarely found in isolation (33, 44). Follicular bronchiolitis and/or LIP are found in around half of the cases reviewed (**Table 1**), and this is also in keeping with a recent review where 20/46 patients had some form of lymphoid infiltration, though not always specified (7).

### Organizing Pneumonia

Organizing pneumonia (OP), intra-alveolar buds of granulation tissue with myofibroblasts and connective tissue, is reported in a substantial number of histological specimens, although to varying degrees between studies (**Table 1**). Cryptogenic organizing pneumonia (COP) is also found in CVID patients and is an important differential diagnosis when OP is the predominant finding on biopsy (40, 44). However, Rao et al. demonstrated the potential for misdiagnosis of CVID ILD when isolated COP was found on limited biopsy samples obtained by bronchoscopy.

OP can have many aetiologies. Larsen et al. reported that in their cohort OP was accompanied by a “dense lymphoid infiltrate”, which was not seen in biopsies from other causes of OP (47). Therefore, in their cohort of 34 patients with CVID and 4 with IgAD, these authors suggest that the combination of these two findings should suggest CVID or IgA deficiency rather than another etiology.

The lack of overlap between OP and pulmonary fibrosis (1/19 cases) in our cases might indicate separate pathological entities; however, significant overlap was described by Rao et al. (11/16 cases) (33, 44), who suggested evolving pathology.

### Pulmonary Fibrosis

Pulmonary fibrosis is described in a quarter of CVID ILD cases (**Table 1**); however, similar to OP, one case series accounts for most of these cases (44), where the majority of patients had some degree of fibrosis. In contrast, Ho et al. found 6.3% of cases where “extensive pulmonary fibrosis” was the “predominant” finding at the time of biopsy; however, it was not reported whether it was a feature in other biopsies to a lesser degree (7).

Interstitial fibrosis in CVID ILD together with lymphoproliferation may resemble some of the patterns of idiopathic interstitial pneumonia, particularly if significant fibrosis (44). Only two studies looked specifically for architectural remodeling, and one of these found this to be associated with significant interstitial fibrosis (33, 44). The presence of fibrosis is a poor prognostic factor; prospective clinical studies are needed to justify earlier treatment (33).

### Immunohistochemistry

**Figure 1:**
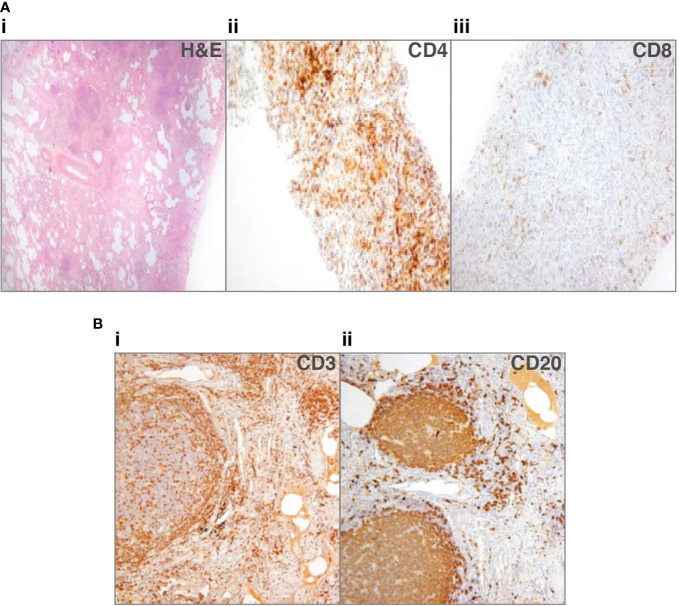
Lung biopsies from patients with common variable immune deficiency (CVID)-related interstitial lung disease (ILD). **(A)** Patient 1: (i) lung biopsy section stained with hematoxylin and eosin (H&E), to show lack of alveolar spaces, and many lymphocytes infiltrating the interstitium (ii) shows staining for CD4^+^ cells that predominate, sometimes in nodules, (iii) shows scanty CD8^+^ cells (33). No granulomata or organizing pneumonia. **(B)** Patient 2: (i) lung biopsy section stained for CD3^+^ cells, showing that T-cells surround follicles and are additionally found in discreet nodules, (ii) shows the follicles to consist of CD20^+^ cells, with only scattered CD20^+^ B-cells in other areas. No granulomata or organizing pneumonia.

Immunohistochemical staining of the lymphocytic infiltrate has produced discordant findings in the cases where it has been performed. CD20^+^ B-cells were found in a small proportion of cases, in follicles with T-cells circumscribing them, although T-cells are also reported more diffusely and in areas without B-cells (28, 33, 44). Rao et al. found a predominance of CD4^+^ T-cells within lymphoid infiltrates and also observed the presence of B-cell follicles surrounded by CD4^+^ T-cells (44). We recently reported a predominance of T-cells in most cases (**Figure 1A**), either CD4^+^ or CD8^+^; only 1 of six had germinal centers within B-cell follicles (**Figure 1B**) (33). Maglione et al. reported actively proliferating germinal centers in some of their patients with B-cell follicles (28). It is important to differentiate these from pulmonary MALToma, as found in two patients in the Oxford series (33).

We suggested that since the predominant T-cells were either CD4^+^ or CD8^+^, this pointed to different pathological entities (33). Chase et al. hypothesized that the inflammatory infiltrate, including B- and T-cells, might contribute to progressive ILD and pulmonary fibrosis, something that therapy directed against B- and T-cells might possibly prevent (40). Similarly, Maglione et al. suggested B-cells may be responsible for leukocyte accumulation in their role as antigen presenting cells and producers of chemokines and/or cytokines, making them a therapeutic target (28).

## Addressing the Heterogeneity of Histopathological Findings CVID-Related ILD

There is a large amount of histopathological heterogeneity in biopsies from CVID-related ILD cases, both from one patient to the next, as well as between different case reports (**Table 1**). We discuss possible reasons for this in respect to the underlying pathophysiology, the patient populations reported, and factors relating to obtaining and interpreting lung biopsies.

### Pathophysiology: A Spectrum of Disease, Separate Diseases, or a Shared Endpoint for Several Diseases?

Since the pathophysiology of CVID ILD is unknown, it is not surprising that there is no explanation for the degree of heterogeneity in the histology (33, 44). CVID-related ILD (or GLILD) was originally defined as a “conglomeration of pulmonary histopathologic abnormalities seen in a subset of patients with CVID (44). The divergent findings may represent a “spectrum” of a single disease (44) or several different pathologies, in addition to the primary antibody deficiency. Another hypothesis is that CVID ILD represents a common “pulmonary reaction pattern” (or “morphological common endpoint”) not only for CVID but also for other PIDs in which similar clinical, radiographical, and histological features have been described (44, 47). None of these hypotheses are mutually exclusive; it may be that the small numbers and the absence of international standardization frustrate the recognition of distinct pathological patterns.

### Patient Populations

Geography may influence the variability observed, with different genetic influences in particular populations. It is interesting that three of the large CVID-related ILD case series, one from the UK and two from the USA, show the most divergence, despite a conscious effort on the part of the former to adhere to similar definitions used previously.

Differences in clinical practice, including diagnosis, cannot be totally discounted. Some series are restricted to patients with spontaneous (non-familial) CVID in adults and others include patients diagnosed in childhood. Since no diagnostic details are given, the exclusion of combined immune deficiencies involving T-cell immunity as well as B-cell failure (5), or known mutations in monogenic disease (e.g. *CTLA4, LRBA, KMT2D, XIAP, RAG1, NFKB1*) (34, 39, 51–57, 63) is unclear.

### Biopsy-Related Factors: Technique, Timing, Treatment, and Interpretation

The method by which a biopsy has been obtained may have a significant impact on the clinical conclusions reached (61). Given that several different biopsy techniques have been used across the cases reported, this may be a contributing factor to some of the variation between cases, though in almost all series so far, imaging was used to obtain the biopsy.

A further consideration is the timing of the biopsy with respect to disease progression but most patients do not undergo repeat biopsies. It is likely that once pulmonary fibrosis and possibly organizing pneumonia are present that these may progress (33).

Another potential contributing factor is whether the biopsy was performed prior to or following corticosteroid or immunosuppressive treatment. These drugs could plausibly alter the patterns observed or mask them entirely, particularly those related to inflammation. While some authors have clearly documented when such drugs were used before biopsies were performed (33), this is not always the case, so firm conclusions cannot be drawn.

In the absence of standardized reporting, reading of the biopsy adds a great deal of potential for variation to be introduced. Although some authors have tried to mirror the approach pioneered by others and/or have a second, independent pathologist review the histology, some degree of both intra- and inter-operator variability is inevitable when faced with an uncommonly encountered pathological entity (33, 40).

## Conclusions and Future Directions

In summary, there is considerable heterogeneity in the histopathological findings both within individual patients, between patients and between study centers, which include lymphoid hyperplasia, granulomata, organizing pneumonia and pulmonary fibrosis. The term “GLILD” is best avoided as not all patients have pulmonary granulomata (32, 46), and its use may mask the histopathological complexity and/or multiple pathological processes (33, 47).

Possible explanations include differences in the timing of sampling with respect to the disease process or treatments, genetic, geographical and environmental factors (7, 33, 44, 47). Finally, inconsistencies in obtaining histological specimens, treated, immuno-stained and described between studies have contributed (33), highlighting an urgent need for standardization of histopathological findings, to allow fairer comparisons to be made between distinct studies. The ability to compare separate studies is of paramount importance when dealing with a rare disease entity.

We need to expand our understanding of the etiology and immunopathogenesis of ILD in CVID, to provide more accurate prognostication and select appropriate treatments. Future studies will incorporate detailed cellular phenotypic, proteomic, transcriptomic and genomic dissection of CVID-ILD, to shed further light on pathogenesis, identify disease-relevant biomarkers and better guide treatment selection.

## Author Contributions

FD and DM prepared the first draft of the manuscript. All authors contributed to editing of subsequent versions and reviewed and authorized the final version. HC and SP played a supervisory role. All authors contributed to the article and approved the submitted version.

## Funding

FD is supported by an Academic Clinical Lectureship from the National Institute for Health Research (NIHR). DM is supported by an Academic Clinical Fellowship from the National Institute for Health Research (NIHR). SP is supported by the NIHR Oxford Biomedical Research Centre and Oxford University Hospitals NHS Foundation Trust.

## Conflict of Interest

The authors declare that the research was conducted in the absence of any commercial or financial relationships that could be construed as a potential conflict of interest.

The handling editor declared a past collaboration with one of the authors, SP.
